# Case Report: A case of occult insulinoma localized by [^18^F] FB (ePEG12)12-exendin-4 positron emission tomography with negative findings of selective arterial calcium stimulation test

**DOI:** 10.3389/fendo.2025.1556813

**Published:** 2025-05-08

**Authors:** Kentaro Sakaki, Takaaki Murakami, Hiroyuki Fujimoto, Yoichi Shimizu, Kanae Kawai Miyake, Daisuke Otani, Shinya Otsuki, Hironori Shimizu, Kazuyuki Nagai, Takumi Nomura, Daisuke Yabe, Yuji Nakamoto, Nobuya Inagaki

**Affiliations:** ^1^ Department of Diabetes, Endocrinology and Nutrition, Graduate School of Medicine, Kyoto University, Kyoto, Japan; ^2^ Radioisotope Research Center, Agency for Health, Safety and Environment, Kyoto University, Kyoto, Japan; ^3^ Department of Diagnostic Imaging and Nuclear Medicine, Graduate School of Medicine, Kyoto University, Kyoto, Japan; ^4^ Department of Diagnostic Pathology, Graduate School of Medicine, Kyoto University, Kyoto, Japan; ^5^ Department of Surgery, Graduate School of Medicine Kyoto University, Kyoto, Japan; ^6^ Department of Diabetes, Shizuoka General Hospital, Shizuoka, Japan; ^7^ Medical Research Institute Kitano Hospital, PIIF Tazuke-kofukai, Osaka, Japan

**Keywords:** exendin-4, PET, insulinoma, GLP-1 receptor, β-cell imaging

## Abstract

**Background:**

Insulinomas, the most common functional pancreatic neuroendocrine tumors, cause hypoglycemia due to excessive insulin production, leading to severe clinical symptoms like coma or death. Resection surgery is the major curative treatment, but preoperative localization is challenging due to their small size. Traditional imaging methods like computed tomography (CT) and magnetic resonance imaging (MRI) often fail to detect tumors, while more invasive procedures like endoscopic ultrasound tissue acquisition (EUS-TA) and the selective arterial calcium stimulation test (SACST), though informative, depend heavily on operator skill and may not always provide conclusive results. There is an urgent need for non-invasive, sensitive localization methods for insulinomas. Glucagon-like peptide 1 receptor (GLP-1R) targeted PET imaging has emerged as a promising tool. We present a clinical case where [^18^F] FB (ePEG12)12-exendin-4 positron emission tomography/CT (^18^F-exendin-4 PET/CT) successfully detected insulinoma, unachievable by conventional imaging, underscoring its potential in guiding minimally invasive surgery.

**Case description:**

A 67-year-old female developed hyperinsulinemic hypoglycemia but could not undergo surgery as conventional imaging methods failed to localize the insulinoma. She was managed with diazoxide for six years, but her symptoms worsened. At 73, she was referred to our hospital. CT, MRI, endoscopic ultrasound, and SACST failed to detect the tumor in any artery. However, ^18^F-exendin-4 PET/CT revealed a nodule with uptake in the dorsal pancreas, suspected to be the culprit lesion. The patient underwent surgery, and although the tumor appeared discontinuous with the pancreas macroscopically, histopathology confirmed it was microscopically continuous, identifying it as a primary pancreatic insulinoma. Post-surgery, she achieved complete remission of symptoms and fully recovered.

**Discussion:**

This case demonstrates the utility of ^18^F-exendin-4 PET/CT, a novel GLP-1 receptor-targeted imaging technique, in accurately localizing an occult insulinoma even with negative findings of SACST, enabling minimally invasive curative surgery.

**Conclusion:**

The ^18^F-exendin-4 PET/CT successfully localized an insulinoma undetectable by other methods, enabling minimally invasive curative resection. This technique offers a valuable diagnostic option for enabling minimally invasive surgery in occult insulinoma cases.

## 1 Background

Insulinomas, the most common functional pancreatic neuroendocrine neoplasms, are observed in 1–4 individuals per million globally (1–4). These tumors cause hypoglycemia provoked by excessive insulin production, which manifests clinically as affected behavior, memory loss, palpitations, tremors, and diaphoresis, potentially resulting in seizures, loss of consciousness, coma, or death (2, 4, 5).

The primary curative treatment option for insulinoma is surgical resection, and precise preoperative localization is crucial for enabling less invasive surgery and minimizing the loss of unaffected pancreatic tissue. Although endoscopic ultrasound-guided radiofrequency ablation (EUS-RFA) has recently been proposed as a potential alternative to surgery in some cases (6), accurate tumor localization remains essential for either approach. Because most insulinomas measure <2 cm in diameter, commonly used imaging modalities such as computed tomography (CT) and magnetic resonance imaging (MRI) are sometimes not sufficient to localize them (7–10). Somatostatin receptor (SSTR)-targeted imaging is also an option; however, it is less sensitive in diagnosing insulinomas compared with other pancreatic neuroendocrine neoplasms (11, 12) and does not provide information on its endocrine function, such as insulin secretion. In these situations, endoscopic ultrasound tissue acquisition (EUS-TA) and the selective arterial calcium stimulation test (SACST) are utilized as they can give insight into the tumor’s endocrine nature. Nonetheless, these procedures are invasive and mostly rely on operator skills (7–10). Additionally, EUS-TA sometimes cannot be performed safely or tissue samples are insufficient to establish a definitive diagnosis (13). Besides, the sensitivity of SACST is not 100%, and SACST sometimes yields negative results despite the tumor’s presence (14, 15).

Therefore, there has been an urgent demand for non-invasive, highly sensitive diagnostic methods for insulinoma, also reflecting the tumor’s insulin secretion function and serving as an alternative option to currently employed diagnostic interventions with significant patient burden (8). Thus, positron emission tomography (PET) targeting the glucagon-like peptide 1 receptor (GLP-1R) has evolved as a noninvasive specific tool for the diagnosis of insulinoma (10, 11, 16).

The importance of accurately localizing insulinoma for the success of minimally invasive surgery has been highlighted, and GLP-1R-targeting imaging is considered a promising approach, especially when other diagnostic tests prove ineffective (10).

Exendin-4 is a 39-amino acid peptide originally isolated from the venom of *Heloderma suspectum*, which shares approximately 53% sequence identity with human glucagon-like peptide-1 (GLP-1) and functions as a GLP-1 receptor agonist (17, 18). Based on its pharmacological profile, exenatide (a synthetic version of exendin-4) was approved by the U.S. Food and Drug Administration (FDA) for the treatment of type 2 diabetes mellitus in 2005 (18).

We recently experienced a case in which [^18^F] FB (ePEG12)12-exendin-4 PET/CT (^18^F-exendin-4 PET/CT) allowed the successful detection of insulinoma, which was not achievable by other multiple conventional imaging tests.

## 2 Case description

A 67-year-old female patient manifested visual disturbances and deteriorated concentration during waking. Her fasting blood glucose level was found to be 32 mg/dL, prompting a visit to the physician. Blood tests after three hours of fasting revealed plasma glucose levels of 32 mg/dL, serum insulin level of 2.5 μU/mL, and serum C-peptide level of 1.26 ng/mL. Following a glucagon stimulation test, the blood glucose levels rose to 80 mg/dL (10 minutes) and 106 mg/dL (30 minutes), thus confirming a diagnosis of hyperinsulinemic hypoglycemia. The test for anti-insulin antibodies was negative, and the other potential causes of hypoglycemia, such as adrenal insufficiency, were ruled out, strongly suggesting insulinoma.

However, no tumor was apparent as contrast-enhanced CT, MRI, or endoscopic ultrasound (EUS) failed to detect any, classifying the case as one with an unknown tumor location. The patient was administered diazoxide at a dose of 125 mg/day. Without any surgical intervention, over the following 6 years, the manifestation of symptoms worsened despite diazoxide therapy, and at the age of 73 years, the patient was directed to our hospital for further examinations.

The patient underwent a hysterectomy for uterine fibroids 30 years ago and was diagnosed with Hashimoto’s disease at the age of 71 years; however, her thyroid functioning parameters were within normal limits. The medication regimen included only diazoxide at a dose of 125 mg four times a day. No alcohol consumption and anti-diabetic medications were recorded in the medical anamnesis.

Upon admission, the patient’s blood pressure was 101/63 mmHg, and the heart rate was 56 bpm. She was 153.5 cm tall and weighed 43.7 kg. There was no change in weight during the last 6 years of illness.

Fasting blood glucose testing performed in the early morning was indicative of hyperinsulinemic hypoglycemia (plasma glucose level of 49 mg/dL, serum insulin of 6.8 μU/mL, and C‐peptide of 1.91 ng/mL). She tested negative for anti-insulin antibodies. Her hemoglobin A1C (HbA1c) level was 5.7%. The levels of adrenocorticotropic hormone, cortisol, thyroid‐stimulating hormone, free thyroxine, and insulin-like growth factor 1 were normal (17.8 pg/mL, 10.1 μg/dL, 3.916 μIU/mL, 1.17 ng/dL, and 71 ng/mL, respectively).

**Figure 1:**
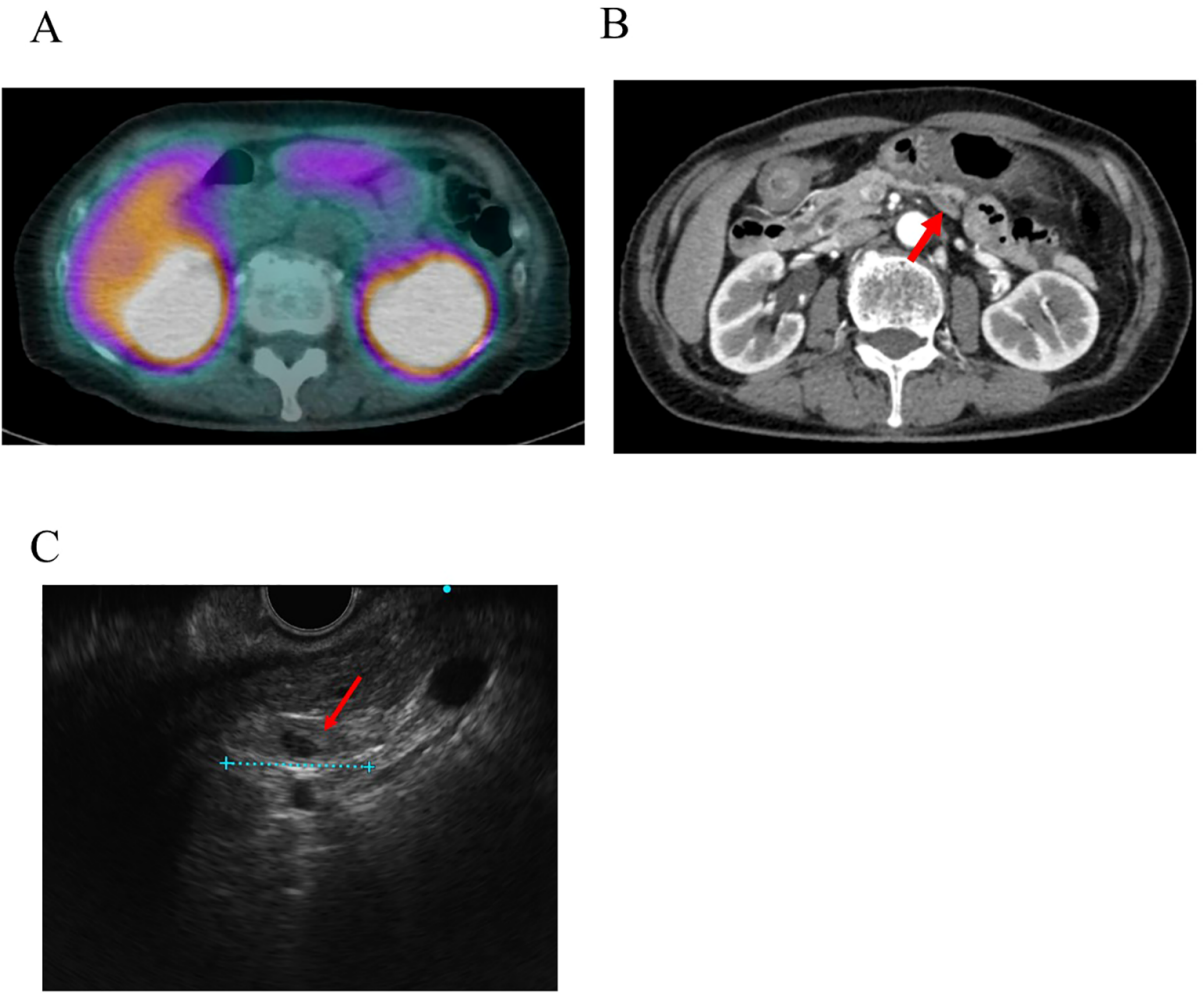
Images produced by conventional techniques. **(A)** [^111^In-DTPA-D-Phe]-octreotide single-photon emission CT (SPECT). No abnormal uptake was observed at the site where the tumor was later identified. **(B)** The nodule identified by [^18^F] FB (ePEG12)12-exendin-4 PET/CT results in contrast-enhanced CT. The red arrow indicates the nodule. **(C)** Nodule detected by endoscopic ultrasound (EUS) conducted after [^18^F] FB (ePEG12)12-exendin-4 PET/CT. The red arrow indicates the nodule.

Contrast-enhanced CT and MRI failed to visualize suspected insulinoma. By EUS, a polycystic lesion was found in the body of the pancreas; however, its morphology was more representative of intraductal papillary mucinous neoplasm, while it was not thought to be the lesion responsible for hypoglycemia. For SSTR-targeted imaging, [^111^In-DTPA-D-Phe]-octreotide single-photon emission CT (SPECT) was performed, and no significant uptake was detected (**Figure 1A**).

Selective arterial calcium stimulation tests (SACST) performed in the superior mesenteric, gastroduodenal, right hepatic, and splenic arteries all failed to demonstrate insulin level increments, which left the tumor’s location undetermined.

**Figure 2:**
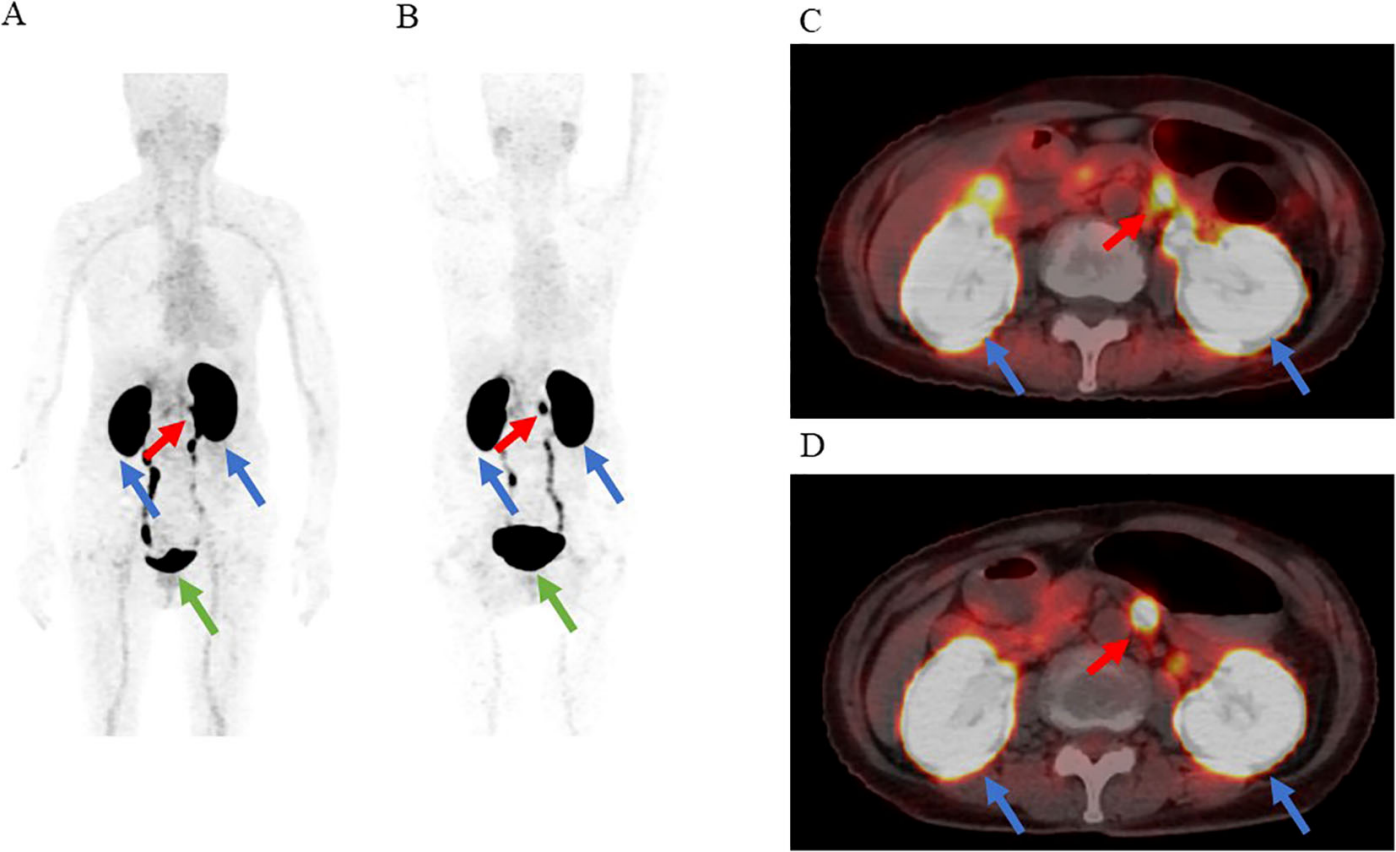
The results of [^18^F] FB (ePEG12)12-exendin-4 positron emission tomography (^18^F-exendin-4 PET)/CT. The red arrow indicates the nodular hyperaccumulation in the dorsal part of the pancreatic body. the blue arrows indicate the kidneys, and the green arrow indicates the bladder. **(A)** Maximum intensity projection of PET 1 h after administration. **(B)** Maximum intensity projection of PET 2 h after administration. **(C)** Fusion imaging of PET/CT 1 h after administration. **(D)** Fusion imaging of PET/CT 2 h after administration.

^18^F-exendin-4 was synthesized as depicted in previous studies (19, 20), and ^18^F-exendin-4 PET/CT scans were performed 1 and 2 hours after intravenous administration of the probe. ^18^F-exendin-4 PET/CT showed nodular hyperaccumulation in the dorsal part of the pancreatic body at both 1 and 2 h after the intravenous probe administration (**Figures 2A–D**). The uptake was remarkably higher than it was in the unaffected pancreatic tissue or those of nearby organs, except for the kidney and gallbladder (**Figures 2A–D**). The nodule signals were easily differentiated from the mentioned structures. No other focal uptakes were observed in the whole body (**Figures 2A, B**). No adverse events were observed during the scans.

In the retrospective evaluation of CT images based on ^18^F-exendin-4 PET/CT findings, we identified an extremely flat nodule on the dorsal side of the pancreas measuring approximately 18 mm in diameter. Initially, based solely on the findings of conventional modalities, the lesion was mistaken for either a normal lymph node, part of the unaffected pancreatic structure, or part of the surrounding connective tissue because of its extremely flat shape and its position appearing to extend outside the pancreatic parenchyma. Consequently, it was not recognized as the culprit lesion. (**Figure 1B**). Subsequent re-evaluations with EUS after the received findings of ^18^F-exendin-4 PET/CT identified an 18 mm hypoechoic, flat structure in the dorsal pancreas (**Figure 1C**). Sonazoid contrast-enhanced ultrasonography captured the lesion before detecting the pancreatic parenchyma. Since the tumor was located on the dorsal side of the pancreas and possibly outside this anatomic structure, it was determined that EUS-TA posed a significant risk of complications and was therefore not conducted. Despite the possible presence of an extrapancreatic lesion or a metastatic lymph node, no other potential culprit lesions were identified by ^18^F-exendin-4 PET/CT, allowing the laparoscopic enucleation of the tumor. The nodule measured 23 × 12 × 2 mm and was extremely flat. Intraoperative macroscopic findings suggested that the tumor’s structure may not have been continuous with that of the pancreas.

**Figure 3:**
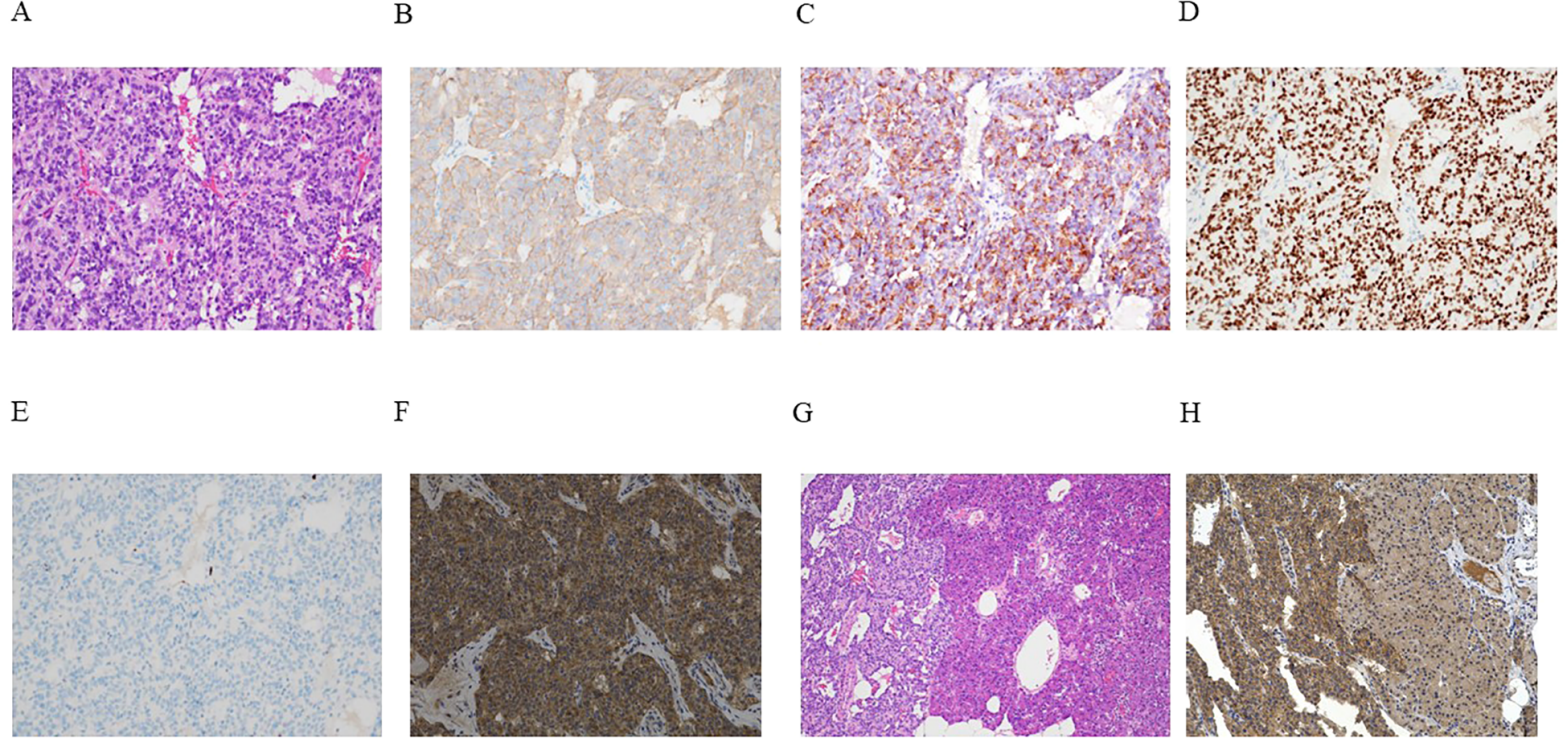
**(A–H)** Outcomes of microscopic analyses of the representative resected tumor. **(A)** Hematoxylin and eosin (HE) staining demonstrated a characteristic pancreatic neuroendocrine tumor manifesting histologically with the trabecular proliferation of cells having oval nuclei and eosinophilic cytoplasm. **(B–G)** Immunohistochemical staining revealed tumor cells that tested positive for synaptophysin **(B)**, insulin **(C)**, and insulinoma-associated protein 1 (INSM1) **(D)**. The Ki-67 labeling index was 0.8% **(E)**. Neoplastic cells also tested positive for the glucagon-like peptide-1 (GLP-1) receptor **(F)**. **(G)** Continuous HE-stained sections of the tumor (left) and unaffected pancreatic parenchyma (right). **(H)** GLP-1 receptor staining showed that GLP-1 receptor expression was more pronounced in the tumor area (left) than in the unaffected pancreatic parenchyma (right). Magnification: ×200 **(A–F, H)**, ×40 **(G)**.

Histopathological analyses performed using hematoxylin and eosin (HE) staining characterized the tumor as a well-differentiated pancreatic neuroendocrine tumor exhibiting trabecular cell proliferation with oval nuclei and eosinophilic cytoplasm (**Figure 3A**). The cells of the nodule tested positive for synaptophysin (**Figure 3B**), insulin (**Figure 3C**), and insulinoma-associated protein 1 (INSM1) (**Figure 3D**). The Ki-67 labeling index was 0.8% (**Figure 3E**). The tumor cells also tested positive for GLP-1 receptors (**Figure 3F**). Although the tumor’s structure seemed to be discontinuous with that of the pancreatic parenchyma during the intraoperative macroscopic evaluation, microscopically, the tumor was continuous with the pancreatic parenchyma, and the insulinoma was identified as a primary pancreatic tumor (**Figures 3G, H**). GLP-1 receptor staining revealed that its expression was more pronounced in the tumor area than in the unaffected pancreatic parenchyma (**Figure 3H**). The tumor was classified as insulinoma, falling under the category of NET G1 according to the WHO (2022) (21).

Following the intervention, the patient’s hypoglycemia and its associated symptoms disappeared. Fasting plasma glucose levels normalized (88 mg/dL), insulin levels changed to 2.8 μU/mL, and C-peptide levels changed to 0.9 ng/mL. Six months after the surgery, hypoglycemia did not recur, and HbA1c levels reached 6.0%. Considering the complication-free postoperative clinical course and the pathological findings, the patient’s complete postoperative recovery was noted.

## 3 Discussion

Patients with insulinoma can develop hyperinsulinemic hypoglycemia; however, there are often diagnostic and treatment delays when conventional techniques fail to localize a tumor, as seen in the reported case (8). In our case, although the tumor appeared slowly progressive, with no apparent enlargement observed over several years following diagnosis, the patient experienced severe hypoglycemia despite diazoxide therapy, resulting in impaired quality of life. This illustrates how such delays in localization and treatment can negatively impact patient well-being.

Some patients cannot undergo surgery and need to be assigned to continuous medical therapy, while others may require extensive surgery that can lead to postoperative diabetes mellitus (8). Hypoglycemia induced by insulinoma can result in coma or even death (2, 4, 5). To prevent such outcomes, it is essential to enable minimally invasive curative surgery (including resection), which would require tumor localization via an accurate, prompt, and non-invasive method that surpasses the diagnostic capacity of conventional examinations (10).

GLP-1 receptor-targeted imaging has been identified as a promising diagnostic technique (10). In the pancreas, GLP-1 receptors are exclusively expressed in β-cells (22), and this is significantly more pronounced in cells of insulinoma compared with normal pancreatic β-cells (16, 23); thus, GLP-1 receptors are considered an ideal target for insulinoma imaging (8, 16). Clinical studies have demonstrated the utility of several exendin-4-based probes for SPECT and PET/CT imaging (11, 16, 24–30). A recent meta-analysis reported that GLP-1 receptor-targeted PET/CT using ^68^Ga-labeled exendin-4 (including ^68^Ga-NODAGA-exendin-4, ^68^Ga-NOTA-exendin-4, and ^68^Ga-DOTA-exendin-4) achieved a sensitivity of 94% and a specificity of 83%, whereas SPECT/CT using ^111^In-DOTA-exendin-4 or ^99m^Tc-EDDA NH2-exendin-4 showed lower diagnostic performance, with a sensitivity of 63% and specificity of 45% (31). However, ^68^Ga-based PET/CT may have limitations such as difficulty in localizing tumors near the kidneys due to physiological renal uptake (32), whereas ^18^F-labeled probes are theoretically expected to provide higher spatial resolution than ^68^Ga-based agents (33). Further accumulation of cases is necessary to assess the diagnostic performance of ^18^F-exendin-4 PET/CT.

We elaborated a novel GLP-1 receptor-targeted imaging method involving a polyethylene glycol (PEG) conjugated (PEGylated) exendin-4-based probe known as ^18^F-exendin-4 (19, 20). ^18^F-exendin-4 PET/CT proved to be highly sensitive and specific in the detection of insulinomas in mice injected with rat insulinoma cells (INS-1 cells), as well as in disease models of mice with multiple insulinomas (19). The probe has been found to be clinically safe in healthy individuals (20). Additionally, clinical cases of insulinoma in which ^18^F-exendin-4 PET/CT enabled the successful detection of this pathologic condition have been detected (24, 25).

In the present case, the tumor was undetectable by CT, MRI, or EUS. It was also not visualized by SSTR-targeted imaging. Although SSTR PET/CT provides higher sensitivity for diagnosis of insulinoma than SSTR SPECT (10, 11), the evaluation was limited to SPECT in this case, as SSTR PET/CT was not available under the national health insurance system in Japan.

SACST also failed to localize the insulinoma, as insulin secretion was not induced by stimulation from any arteries. The non-response of the insulinoma to SACST in this case may be attributed to its unique vascular supply due to its protruding location or an incidental low expression of calcium sensing receptor. After the result of ^18^F-exendin-4 PET/CT was confirmed, the patient was reexamined and the nodule could be detected by EUS, but due to the location of the tumor, EUS-TA could not be performed. However, the information obtained from PET/CT provided accurate localization and led to curative surgery by enucleation.

SACST and GLP-1 receptor-targeted PET/CT are based on fundamentally different physiological mechanisms. SACST assesses insulin secretion in response to calcium stimulation via vascular routes, while GLP-1 receptor imaging reflects receptor expression in tumor tissue. Given these differences, the two modalities may be complementary rather than mutually exclusive. Notably, the present case represents a rare instance in which SACST failed to localize the tumor, whereas GLP-1 receptor-targeted imaging successfully did so, supporting the potential utility of this modality even in SACST-negative cases.

In previous reports of the other GLP-1 receptor-targeted imaging, several cases have been documented in which tumors undetectable by CT or MRI were successfully localized (27–30). However, in most of these cases, the patients were either positive on SACST or did not undergo SACST evaluation (27–30). Therefore, the clinical information regarding the usefulness of GLP-1 receptor-targeted imaging in comparison with SACST is still lacking and the finding of GLP-1 receptor-targeted imaging in cases with SACST-negative is rare. As for ^111^In-DOTA-exendin-4 SPECT/CT, among six insulinoma cases diagnosed, three did not undergo SACST and the other three (including a case of ectopic insulinoma) were all positive in SACST (27). In a multicenter study using ^111^In-DTPA-exendin-4 SPECT/CT, only seven out of 25 cases underwent SACST; although two of these were false-positive, none were false-negative (28). In the report that verified the diagnostic ability of ^68^Ga-NOTA-Exendin-4, there was no mention of comparison with the results of SACST (29). Notably, in a report comparing ^68^Ga-DOTA-exendin-4 PET/CT and ^111^In-DOTA-exendin-4 SPECT/CT, 10 out of 52 participants underwent SACST. Among these, only three cases were identified where tumors were detected by ^68^Ga-DOTA-exendin-4 PET/CT and confirmed as insulinoma in the final pathological diagnosis, despite being false-negative in SACST (30). The summary of the SACST-false-negative cases is shown in **Table 1**.

**Table 1:** Comparison of clinical characteristics and results of conventional tests of previously reported occult insulinoma cases and the present case.

Clinical Characteristics and Test Results	Patient 1 (Ref:30)	Patient 2 (Ref:30)	Patient 24 (Ref:30)	Present Case
Duration of symptoms (months)	20	39	120	72
Computed tomography	Negative	NA	Negative	Negative
Magnetic resonance imaging	Negative	Negative	Negative	Negative
Endoscopic ultrasound	Negative	Negative	Negative	Negative
Selective arterial calcium stimulation test	Negative	Negative	Negative	Negative
^111^In-DOTA-exendin-4 SPECT/CT	Negative	Positive	Positive	
^68^Ga-DOTA-exendin-4 PET/CT	Positive	Positive	Positive	
[^18^F] FB (ePEG12)12-exendin-4 PET/CT				Positive
Surgical Procedure	Enucleation	Pancreas tail resection	Body resection + pancreatico-jejunostomy	Enucleation
Dimension (mm)	7	12	15	18
Localization	Tail	Tail	Body	Body
Final pathological diagnosis,	Insulinoma	Insulinoma	Insulinoma	Insulinoma

Patient numbers are consistent with those used in the original publication (Ref:30 in the main text of this manuscript). Individual patient age and sex were not provided in Ref:30 and are therefore not included in this table. NA, not applicable.

In addition, endoscopic ultrasound can provide high-resolution anatomical information, including the spatial relationship between the tumor and the main pancreatic duct, which is useful for determining the appropriate surgical approach (34). These facts underscore the importance of individualized strategies for diagnosis and preoperative evaluation with appropriate understanding of complementary roles of each modality. To date, however, no studies have systematically evaluated the diagnostic accuracy or clinical impact of combining GLP-1 receptor-targeted imaging and EUS. Further investigations are warranted to establish optimized multimodal approaches for the localization and management of insulinoma.

The present case is the first case in which ^18^F-exendin-4 PET/CT successfully localized an occult insulinoma that remained elusive by CT, MRI, EUS, and even SACST. Even with all GLP-1 receptor-targeted imaging, only a few cases demonstrated that GLP-1 receptor-targeted imaging can localize occult insulinoma that was false-negative in SACST. This case highlights both the critical importance of precise tumor localization in optimizing the treatment of insulinoma and the utility of ^18^F-exendin-4 PET/CT, even in the most challenging clinical scenarios including negative findings of SACST.

## 4 Conclusion

The ^18^F-exendin-4 PET/CT method provided comprehensive localization information for the insulinoma that could not be detected by other techniques and finally enabled the minimally invasive curative resection of the neoplasm. Thus, ^18^F-exendin-4 PET/CT could be a promising diagnostic option that facilitates a minimally invasive approach in the surgical treatment of insulinomas.

## Data Availability

The original contributions presented in the study are included in the article/supplementary material. Further inquiries can be directed to the corresponding author.
